# Persistent *Cryptosporidium parvum* Infection Leads to the Development of the Tumor Microenvironment in an Experimental Mouse Model: Results of a Microarray Approach

**DOI:** 10.3390/microorganisms9122569

**Published:** 2021-12-12

**Authors:** Manasi Sawant, Sadia Benamrouz-Vanneste, Anthony Mouray, Peggy Bouquet, Nausicaa Gantois, Colette Creusy, Erika Duval, Adriana Mihalache, Pierre Gosset, Magali Chabé, David Hot, Eric Viscogliosi, Gabriela Certad

**Affiliations:** 1 U1019-UMR 9017-CIIL-Centre d’Infection et d’Immunité de Lille, Institut Pasteur de Lille, Université de Lille, CNRS, Inserm, CHU Lille, F-59000 Lille, France; manasi.sawant@pasteur-lille.fr (M.S.); sadia.benamrouz@univ-catholille.fr (S.B.-V.); nausicaa.gantois@pasteur-lille.fr (N.G.); magali.chabe@univ-lille.fr (M.C.); eric.viscogliosi@pasteur-lille.fr (E.V.); 2 Laboratoire Ecologie et Biodiversité, Unité Smart and Sustainable Cities, Université Catholique de Lille, F-59000 Lille, France; 3 Plateforme d’Expérimentations et de Hautes Technologies Animales, Institut Pasteur de Lille, F-59000 Lille, France; anthony.mouray@pasteur-lille.fr; 4 Institut Pasteur de Lille, US 41-UMS 2014-PLBS, Université Lille, CNRS, Inserm, CHU Lille, F-59000 Lille, France; Peggy.Bouquet@pasteur-lille.fr (P.B.); david.hot@pasteur-lille.fr (D.H.); 5 Service d’Anatomie et de Cytologie Pathologiques, Groupement des Hôpitaux de l’Institut Catholique de Lille (GHICL), F-59000 Lille, France; creusy.colette@ghicl.net (C.C.); duval.erika@ghicl.net (E.D.); mihalache.adriana@ghicl.net (A.M.); gosset.pierre@ghicl.net (P.G.); 6 Délégation à la Recherche Clinique et à l’Innovation, Groupement des Hôpitaux de l’Institut Catholique de Lille, F-59462 Lomme, France

**Keywords:** Apicomplexa, *Cryptosporidium*, animal model, transcriptome, anti-microbial peptides, α-defensins, inflammation, tumor microenvironment, colon cancer

## Abstract

*Cryptosporidium* spp. are enteric protozoa parasites that infect a variety of vertebrate hosts. These parasites are capable of inducing life-threatening gastrointestinal disease in immunocompromised individuals. With the rising epidemiological evidence of the occurrence of *Cryptosporidium* infections in humans with digestive cancer, the tumorigenic potential of the parasite has been speculated. In this regard, *Cryptosporidium parvum* has been reported to induce digestive adenocarcinoma in a rodent model of chronic cryptosporidiosis. However, the processes by which the parasite could induce this carcinogenesis are still unknown. Therefore, the transcriptomes of *C. parvum* infected ileo-cecal regions of mice developing tumors were analyzed in the current study. For the first time, downregulation of the expression of α-defensin, an anti-microbial target of the parasite in response to *C. parvum* infection was observed in the transformed tissues. This phenomenon has been speculated to be the result of resistance of *C. parvum* to the host defense through the upregulated expression of interferon γ-stimulated genes. The inflammatory response generated as result of attenuated expression of anti-microbial peptides highlights the role of immune evasion in the *C. parvum*-induced tumorigenesis. The study has also succeeded in the characterization of the tumor microenvironment (TME) which is characterized by the presence of cancer associated fibroblasts, myeloid-derived suppressor cells, tumor-associated macrophages and extracellular matrix components. Identification of immune suppressor cells and accumulation of pro-inflammatory mediators speculates that chronic inflammation induced by persistent *C. parvum* infection assists in development of an immunosuppressive tumor microenvironment.

## 1. Introduction

The Apicomplexan parasite *Cryptosporidium* is recognized as one of the main waterborne agents causing diarrhea worldwide. This ubiquitous intracellular parasite is responsible of self-limited diarrhea in immunocompetent individuals but is capable of causing life-threatening disease in those who are immunocompromised [1]. Different cohort studies have reported that *Cryptosporidium* is one of the main pathogens responsible for severe diarrhea and mortality in children under 5 years old [2,3]. In addition, more than 200 outbreaks have been reported worldwide due to contaminated recreational or drinking water [4,5,6]. The low number of parasites required for an infection [7] coupled with the well-known resistance of *Cryptosporidium* oocysts to disinfection methods facilitates the waterborne transmission of cryptosporidiosis [8,9]. As a result, an ever-growing number of persons could be exposed to this parasite around the world. However, despite its prevalence and impact on public health, neither treatment nor vaccine against *Cryptosporidium*, are yet available [10].

Strikingly, *Cryptosporidium* has been identified with a significant higher prevalence among Lebanese [11], Chinese [12], and Polish [13] patients with colon cancer before undergoing any oncological treatment. The prevalence of *Cryptosporidium spp.* in colon cancer patients has been mainly restricted to *C. parvum* [11,12,13] and *C. hominis* [11]. Some additional reports showed that immunocompromised individuals suffering from HIV infection and cryptosporidiosis are at high risk of colorectal cancer (CRC) compared to HIV patients without cryptosporidiosis [14,15], indirectly suggesting that *Cryptosporidium* infection might somehow be associated with development of digestive cancer.

This causal link between *C. parvum* infection and cancer has been explored in an experimental model of cryptosporidiosis. In this severe combined immunodeficiency (SCID) rodent model, *C. parvum* infection was able to induce ileo-cecal adenocarcinoma [16,17,18]. The carcinogenic potential of this parasite was also confirmed in enteric explants [19]. The *C. parvum*-induced transformation did not exhibit classical canonical trademarks of colon cancer [18]. In contrast, alterations in cellular expression of APC and β-catenin were reported along with prominent basolateral and cytoplasmic localization of β-catenin potentially correlated with the involvement of a non-canonical Wnt pathway [18].

In parallel, inflammatory monocytes recruited at the sub-epithelial spaces were reported to assist *C. parvum* in reducing the transepithelial resistance via delocalization of E-cadherin and β-catenin from the adherens junctions of intestinal epithelial cells (IECs) [20]. Thus, inflammatory response generated by the infection could potentially disrupt epithelial barrier function and eventually result in chronic inflammation which could promote tumorigenesis. In this respect, activation of pro-inflammatory signaling pathway such as nuclear factor-kappa B (NF-κβ) by *C. parvum* has been shown to inhibit apoptosis of infected epithelial cells [21].

Interestingly, a mouse model of inflammation inducing carcinogenesis with deletion of MCC (mutated in colorectal cancer) gene, demonstrated the absence of hyperactivation of β-catenin pathway [22] likewise to *C. parvum* associated cancer in which β-catenin was not found in the nucleus of the transformed cells [18]. This study emphasized an example of inflammation inducing DNA damage in the absence of external carcinogens. Hence, observation of *C. parvum* infection driven cancer without recording the DNA damage caused by canonical mutations [18] suggests that the parasite can be regarded as an external carcinogen, which might contribute to chronic inflammation during the infection, leading to tumor development [18].

Several microarray data are available wherein the gene profiles of *C. parvum* infected versus uninfected cells or tissue have been compared. These studies have identified cancer related pathways such as Hedgehog [23], Wnt [23] and p38/MAPK [24] to be altered by the parasite for its survival. Considering that these studies were carried out in already transformed cells, it was difficult to categorize these alterations as to play a significant role in *C. parvum* infection-induced cancer development. In the current transcriptomics study, we aimed to decipher novel molecules and signaling pathways which could be considered as potential specific markers of *C. parvum* infection induced digestive cancer.

## 2. Materials and Methods

### 2.1. Cryptosporidium Oocysts

*C. parvum* strain IOWA oocysts were purchased from Waterborne, Inc. (New Orleans, LA, USA) and stored in phosphate-buffered saline (PBS) supplemented with penicillin, streptomycin, gentamycin, amphotericin B and 0.001% Tween 20 at 4 °C until use. Absence of bacteria and fungi was assured by testing the oocyst suspensions on Plate Count Agar and Sabouraud plates for 1 week at 37 °C. The oocyst viability was assessed as described previously [19]. The oocyst suspension was first loaded on the FAST READ 102^®^ slide (Biosigma, Cona, Italy) and observed under a Zeiss optical microscope (Zeiss, Oberkochen, Germany) to determine the number of intact and empty oocysts. The viability of oocysts was subsequently calculated by determining the ratio between the number of empty oocysts and the total number of intact and empty oocysts × 100.

### 2.2. Mouse Model of Cryptosporidiosis

Twenty-four seven-week-old CB17-SCID mice were obtained from a colony bred and regularly controlled for infections (including *Helicobacter* spp.) at the Pasteur Institute of Lille (France). Animals were maintained under aseptic conditions in an isolator during the whole experimentation, in a 12 h light-dark cycle with water and a standard diet (65% carbohydrate, 11% fat, and 24% protein; SAFE, Augy, France). Mice were administered with 4 mg/L of dexamethasone (Merck, Lyon, France) through drinking water. Dexamethasone treatment started 2 weeks prior to inoculation with the parasite and maintained during the entire experimentation. Dexamethasone-containing water was replaced three times a week. Infective doses of *C. parvum* (10^5^ oocysts/mouse) were prepared and inoculated by intra-gastric feeding. Control animals were inoculated with PBS. Assessment of the clinical conditions of the mice was performed regularly to detect and then minimize suffering. Clinical signs that could constitute an endpoint included, but were not limited to, rapid or progressive weight loss, debilitating diarrhea, rough hair coat, hunched posture, lethargy or any condition interfering with daily activities (e.g., eating or drinking, ambulation or elimination). During the course of the experiment, 1 uninfected and 1 infected mice were found dead. The experiment was pursued with the remaining 22 mice which were distributed into 4 groups as follows: Group A (one uninfected mouse and two infected mice at 45 day post-infection (PI) for histological analysis), Group B (one uninfected mouse and one infected mouse at 93 day PI), Group C (four uninfected mice and five infected mice at 45 day PI for microarray analysis), Group D (three uninfected mice and five infected mice at 93 day PI for microarray analysis). Mice were euthanized by carbon dioxide inhalation and samples from the ileo-cecal region were collected. At 45 day PI, entire ileo-cecal region was retrieved whereas at 93 day PI, polypoid masses visible macroscopically and measuring approximately 2.5 mm in diameter were harvested. Experiments were conducted in the animal facility at the Institute Pasteur of Lille (research accreditation number, D 59 350 009). Animal protocols were approved by the French regional ethical committee with the number APAFIS#9621.

### 2.3. Oocyst Shedding

The oocyst shedding was evaluated by collecting freshly excreted fecal pellets from each mouse at the time of euthanasia. Total genomic DNA was extracted from 200 mg of feces by using the QIAamp Fast DNA Stool Mini Kit (Qiagen GmbH, Hilden, Germany) according to the manufacturer’s protocol. The presence of parasite was detected by using the TaqMan real time PCR (qPCR) assay as previously described [25]. Briefly, the assay amplified a DNA fragment located in the 18S rRNA gene locus (GenBank accession no. EU675853, positions 33 to 211). The forward (5′-CATGGATAACCGTGGTAAT-3′) and reverse (5′-TACCCTACCGTCTAAACTG-3′) primers were designed to amplify a 178 bp fragment. A TaqMan probe homologous to a conserved region of the sequence (Pan-crypto probe; FAM-CTAGAGCTAATACATGCGAAAAAA-MGB-BHQ [FAM, 6-carboxy-fluorescein; MGB, minor-groove-binding ligand; BHQ, black hole quencher]) was designed to detect *Cryptosporidium*. The qPCR reactions were performed on a Rotor-Gene 6000 instrument (Corbett Research, Qiagen) with 50 ng of extracted DNA, 200 nM of each primer, 100 nM of the probe and 1X of Light Cycler 480 Probes Master. The gene amplification consisted of activation of the *Taq* DNA polymerase for 10 min at 94 °C, followed by 45 cycles of 94 °C for 10 s, 54 °C for 30 s and 72 °C for 10 s. Fluorescence signal was acquired at end of elongation step and the amplification data were analyzed using Rotor-gene Q Series software. The results were displayed using GraphPad Prism (version 9.1.0 (221), latest update on 2021/03/15)

### 2.4. Histological Examination

Ileo-cecal regions of mice from groups A and B were recovered then fixed in 4% buffered formalin. Formalin-fixed and paraffin-embedded specimens were sectioned at a thickened of 4 μm, stained with hematoxylin, eosin and saffron and examined microscopically for the detection of histological modifications of the host tissue. Pathological changes found in the mouse caecum were classified according to the Vienna classification of tumors of the digestive system in humans [26] taking into account the nomenclature for histological assessment of intestinal tumors in rodent models [27] as previously described [28].

### 2.5. Agilent Microarray Analysis

The SurePrint G3 Mouse GE 8x60K Microarray and services (Agilent Technologies, Santa Clara, CA, USA) were used to process the samples and perform genome-wide analysis. Briefly, at specific time points, the individual mice from groups C and D were euthanized and the caecum tissue was harvested then placed in four volumes of RNAlater (Qiagen, Valencia, CA, USA) before storage at −80 °C. Total RNA was isolated from tissue using TRIzol™ Reagent (Invitrogen, Carlsbad, CA, USA) and treated with DNAse I (Sigma Life Sciences, St. Louis, MO, USA) according to manufacturer’s protocol. RNA quality and quantity were determined using Agilent RNA6000 Nano kit by capillary electrophoresis and Agilent 2100 Bioanalyzer (Agilent Technologies). Total RNA (100 ng) from each sample was labelled as described in One color microarray based gene expression analysis protocol (Agilent Technologies) using the Agilent Quick-Amp Labeling kit according to the manufacturer’s instructions. After purification using a RNeasy Mini Kit (Qiagen), cRNA yield and incorporation efficiency (specific activity) into the cRNA were determined using a NanoDrop 2000 spectrophotometer (Thermo Fisher Scientific, Waltham, MA, USA). For each sample, a total of 600 ng of cRNA was fragmented and hybridized overnight at 65 °C. After hybridization, slides were washed before being scanned on a SureScan Microarray Scanner (Agilent Technologies) and further processed using Feature Extraction v10.7.3.1 software. The resulting text files were uploaded into language R v4.0.3 and analyzed using the Linear Model for Microarray Data (LIMMA) package [29,30]. A ‘within-array’ normalization was performed using LOWESS (locally weighted linear regression) to correct for dye and spatial effects [31]. Moderate t-statistic with empirical Bayes shrinkage of the standard errors [32] was then used to determine significantly modulated genes. Statistics were corrected for multiple testing using a false-discovery rate approach.

### 2.6. Data Analysis

To visualize and explore the molecular interaction networks of the differentially expressed genes, the subsequent data were uploaded into the Ingenuity Pathways Analysis (IPA) software (QIAGEN CLC Genomics Workbench 20.0; version 68752261; latest update on 6 September 2021) (https://digitalinsights.qiagen.com/) (Qiagen, Redwood City, CA, USA) to organize the differentially expressed genes into networks based on the Ingenuity Knowledge Database (IKB), an extensive database of functional direct and indirect interactions between genes from peer-reviewed publications. Particularly, the present study applied IPA system to uncover the signaling pathways, interactions and functional roles associated with differentially expressed genes in *C. parvum* infected caecum tissues in comparison with controls. IPA uses a network generation algorithm to segment the network map between molecules into multiple networks and assign scores for each network. The score is generated based on hypergeometric distribution, where the negative logarithm of the significance level is obtained by Fisher’s exact test at the right tail [33]. For upstream regulators, disease and function, the −log (*p*-value) > 4 was taken as threshold, the z-score > 2 was defined as the threshold of significant activation, whilst z-score < −2 was defined as the threshold of significant inhibition. For upstream regulators, the *p*-value of overlap < 0.05 was also set as the threshold. For regulator effects, consistency scores were calculated, with a high consistency score indicating accurate results for the regulatory effects analysis. The algorithm used for calculating the z-scores and *p*-values of overlap has been described previously [34]. The graphs were produced used GraphPad Prism (version 9.1.0 (221), latest update on 15 March 2021).

### 2.7. Validation by Quantitative Reverse Transcription PCR

Quantitative Reverse Transcription PCR (RT-qPCR) was used to validate microarray results of four upregulated and two downregulated genes. cDNA was synthesized from 1 μg of total RNA extracted from the caecum tissue using oligo-(dT)_20_ primer and Superscript III reverse transcriptase (RT) in a 20 μL reaction (Invitrogen) according to the manufacturer’s instruction. RT-qPCR was performed with a Corbett Research RG-6000 Real time PCR machine (Qiagen). Primers used are listed in supplementary Table S1 and glyceraldehyde 3-phosphate dehydrogenase (GAPDH) was used as the endogenous control. Each RT-qPCR reaction (20 µL) contained 7.5 µL Brilliant III Ultra-Fast qPCR mastermix (Agilent Technologies), 400 nM of each forward and reverse primers and 50 ng of template cDNA. PCR amplification consisted of activation of the *Taq* DNA polymerase for 3 min at 95 °C, followed by 40 cycles of 95 °C for 10 s, 57 °C for 20 s and 72 °C for 20 s. Fluorescence signal was acquired at the end of elongation step and the amplification data were analyzed using Rotor-gene Q series software. The 2^−ΔΔCt^ method was used to calculate the relative expression levels of target genes in infected mice with the constitutively expressed endogenous control and the ΔC_t_ value of the uninfected mice was used as the calibrator [35]. The results were displayed using GraphPad Prism (version 9.1.0 (221), latest update on 15 March 2021).

## 3. Results

### 3.1. C. parvum Induces Chronic Infection and Neoplasia in a Mouse Model

**Figure 1:**
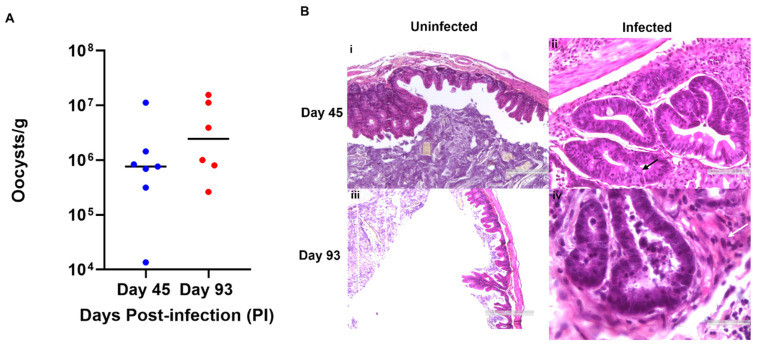
Experimental SCID mouse model of chronic cryptosporidiosis and neoplasia. (**A**) Pattern of oocyst shedding (oocysts/g of feces) in mice at days 45 (blue dots) and 93 PI (red dots) wherein each dot represents individual mouse. The line in each pattern corresponds to the geometric mean of oocyst shedding per group. (**B**) Histological examination of *C. parvum* uninfected (i–iii) and infected (ii–iv) ileo-cecal regions at days 45 and 93 PI. Presence of significant cytonuclear atypia and appearance of intra-mucosal adenocarcinoma are observed at day 45 PI (black arrow). Desmoplastic reaction is also reported around the invasive neoplastic glands (white arrow) at day 93 PI.

Using the well-documented model of intestinal cryptosporidiosis (SCID mice treated with dexamethasone) through the oral administration of the oocysts, infection of *C. parvum* was detected by quantification of the oocyst shedding (Figure 1A). Upon histological examination of the ileo-cecal region (Figure 1B), well-differentiated adenocarcinoma processes invading the submucosae were observed in two out of seven mice that were euthanized at day 45 PI. Glands with necrotic content and a large number of polymorphic inflammatory elements were also observed. Mice euthanized at day 93 PI presented polypoid, sessile, masses, measuring approximately 2.5 mm in diameter in the ileocecal region. At histological examination, we observed an invasive well-differentiated adenocarcinomas that progressed into the lamina propria (intramucosal carcinoma, category 4.4 Vienna classification) in one out of six and in the other five a well-differentiated adenocarcinoma penetrating the inner layer of the muscularis (category 5 Vienna classification) with a desmoplastic response around the glands, which is a typical feature of invasive carcinoma in rodents. Necrosis of the glands and numerous polymorphic inflammatory elements in the chorion were also described. Presence of numerous parasites at different developmental stages in the lumen of the glands were also reported at days 45 and 93 PI.

### 3.2. Chronic Cryptosporidiosis Induced Neoplasia Results in Global Gene Profile Alterations

The gene expression profiles were significantly altered with 43 and 931 genes reported to be differentially regulated at day 45 PI and day 93 PI, respectively (LogFC2.0; adj *p*-value < 0.05) as shown in Table 1. The entire lists of regulated genes are provided as Supplementary Tables S2 and S3.

Fold changes of infected mice at day 45 PI and day 93 PI were compared with those of control uninfected animals of matching genotype with significant adj *p*-value (<0.05).

The experimental datasets from each time point were subjected to core analysis using IPA software. Analysis was performed to characterize the global functions associated with the altered gene profiles. As a result, 37 genes at day 45 PI and 900 genes at day 93 PI were mapped using the software. The software predicted 35 genes to be “analysis-ready molecules” at day 45 PI wherein 33 of them were predicted as upregulated and the two remaining ones as downregulated. At day 93 PI, 758 “analysis-ready molecules” were predicted wherein 566 and 192 genes were predicted to be upregulated and downregulated, respectively.

**Figure 2:**
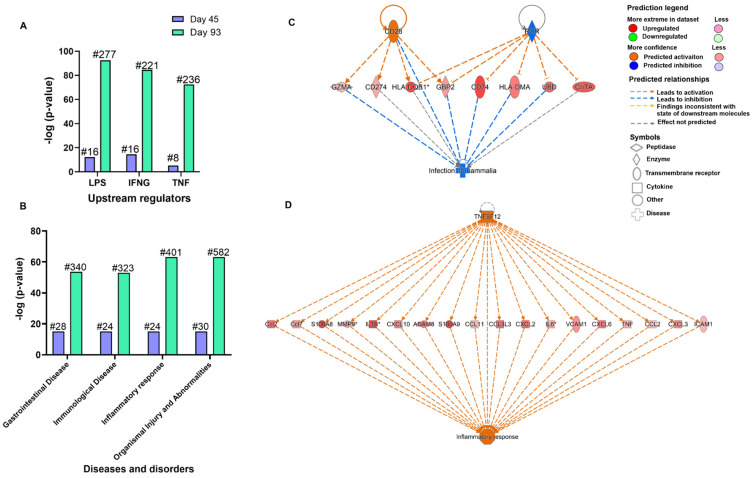
IPA analysis of transcriptomes of *C. parvum* infected caecum tissue when compared to uninfected tissue at days 45 and 93 PI. (**A**) Top three common upstream regulators observed at days 45 and 93 PI are represented in terms of −log (*p*-value) on y-axis. (**B**) Top four common diseases and disorders observed at days 45 and 93 PI represented in terms of −log (*p*-value) on y-axis (# = no. of differentially expressed genes). (**C**) Molecular network diagram representing the top regulatory pathways predicted to be involved in the function mammalian infection at day 45 PI with a consistency score of 2.121. (*) Indicates that multiple identifiers are present in the dataset file which map to a single gene in the global molecular network. (**D**) Molecular network diagram representing the top regulatory pathways predicted to be involved in inflammatory response at day 93 PI with a consistency score of 4.213.

As a part of core analysis, the experimental datasets were also subjected to different features of the software which included “upstream regulators”, “diseases and functions” and “regulatory effects” analysis. The upstream regulators are the predicted molecules involved in the regulation of gene expression changes within the datasets. By applying the threshold of overlap *p*-value < 0.05, a total of 41 and 306 upstream regulators were identified at day 45 PI and day 93 PI, respectively. The overlap *p*-value measures whether a statistically significant overlap is pointed out between the dataset genes and the genes that are regulated by the transcriptional regulator. Among them, 29 (activated) had activation z-score > 2 and 12 (inhibited) had activation z-score < −2 at day 45 PI whereas about 140 upstream regulators were predicted to be activated and about 166 inhibited at day 93 PI. The top three upstream regulators identified at both time points were Lipopolysaccharide (*LPS*), Interferon-γ (*IFNγ*) and Tumor necrosis factor (*TNF*) (Figure 2A). *IFNγ* was observed to be the most powerful activator at day 45 PI (Z-score = 3.954, overlap *p*-value = 2.97 × 10^−15^, 16 target molecules) whereas *LPS* was identified as the most powerful activator at day 93 PI (Z-score = 11.165, overlap *p*-value = 3.23 × 10^−93^, 277 target molecules). Further, by applying the −log (*p*-value) > 4 threshold, the top diseases and cellular functions associated with the molecules in the datasets were determined. As shown in Figure 2B, the top four “Diseases and Disorders” observed to be common at both time points were as follows: “Gastrointestinal Disease” [day 45 PI (*p*-value = 8.06 × 10^−16^), day 93 PI (*p*-value = 1.90 × 10^−54^)], “Immunological Disease” [day 45 PI (*p*-value = 8.04 × 10^−16^), day 93 PI (*p*-value = 4.11 × 10^−68^)], “Inflammatory Response” [day 45 PI (*p*-value = 1.61 × 10^−21^), day 93 PI (*p*-value = 8.61 × 10^−64^)] followed by “Organismal Injury and Abnormalities” [day 45 PI (*p*-value = 1.06 × 10^−23^), day 93 PI (*p*-value = 8.61 × 10^−64^)]. Out of the top four, “Organismal Injury and Abnormalities” was reported to have highest number of molecules to be involved from each dataset (day 45 PI = 30 molecules and day 93 PI = 582 molecules). Moreover, regulatory effects analysis algorithm was used to connect the upstream regulators, dataset molecules and downstream disease and functions to generate a hypothesis of how a particular function is regulated in the dataset. The regulator effects are determined in terms of a consistency score which is a measure of how casually consistent and densely connected a regulatory network is. The upstream regulators and diseases and functions included in the analysis were of −log (*p*-value) > 4 and z-score > 2. As a result of this analysis, at day 45 PI the highest ranked regulators were observed to be *CD28* and cytochrome p450 oxidoreductase (*POR*) with a consistency score of 2.121 which may be involved in the inhibition of cellular function of mammalian infection mainly through mediating their targets which encode regulatory proteins such as *UBD* (ubiquitin D), serine proteases such as Granzyme A (*GZMA*), IFNγ-induced GTPases such as Guanylate binding protein 2 (*GBP2*) and molecules associated with major histocompatibility complex (MHC) class II such as *CD74*, *HLA-DQB1*, *HLA-DMA* and *CIITA* (Figure 2C). At day 93 PI, Tumor necrosis factor ligand superfamily member 12 (*TNFSF12*), also known as TNF-related weak inducer of apoptosis (TWEAK), was observed with a highest consistency score of 4.243 to be involved in the activation of cellular function of inflammatory response via mediating the targets such as matrix metalloproteinases (*MMP*, *ADAM8*), chemokines and cytokines (*CCL7*, *CXCL10, CCL11*, *CCL3L3*, *CXCL2*, *CXCL6*, *CCL2*, *CXCL3*, *S100A8*, *S100A9*, *IL1β*, *IL6* and *TNF*) and immunoglobulins (*ICAM1* and *VCAM1*) (Figure 2D).

### 3.3. Regulation of Anti-Microbial Peptides Like α-Defensins during the Course of C. parvum Infection

**Figure 3:**
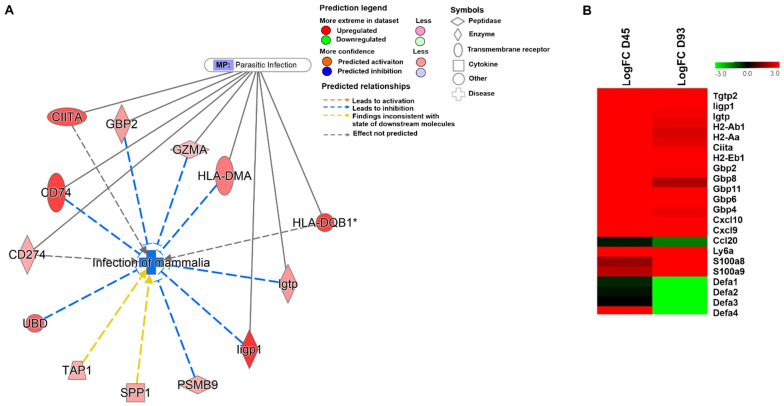
Analysis of molecules regulated during *C. parvum* infection. (**A**) Molecular network representing genes involved in mammalian infection overlaid with the function of parasitic infection. (*) Indicates that multiple identifiers are present in the dataset file which map to a single gene in the global molecular network. (**B**) Heatmap of genes significantly upregulated or downregulated in infected caecum tissue compared to uninfected tissue at days 45 and 93 PI.

As previously described, mammalian infection was observed to be a highly downregulated function at day 45 PI (z score = −2.017). Parasitic infection was just following the mammalian infection function with a z-score of −1.969. Out of the 13 molecules identified to be involved in mammalian infection, eight of them were in common with the parasitic infection function (Figure 3A). Interestingly, the expression levels of some highly regulated genes predicted to inhibit parasitic infection which include antigen representing MHC class II molecules (*H2-AB1*, *H2-AA*, *CIITA* and *H2-EB1*), IFNγ induced GTPases (*IIGP1*, *IGTP*, *GBP2*, *GBP8*, *GBP11*, *GBP6* and *GBP4)* and chemokines *(CXCL10* and *CXCL9)* does not differ between day 45 PI and day 93 PI (Figure 3B). At day 93 PI, the experimental dataset revealed downregulation of the chemokine *CCL20*, known to exert anti-microbial activity against *C. parvum* (Figure 3B). The analysis also identified α-defensins 4 (*DEFA4*), an anti-microbial peptide, to be highly upregulated at day 45 PI (Log FC = 6.37) compared to day 93 PI (Log FC = −6.07) wherein it was highly downregulated (Figure 3B). Other isoforms of α-defensins, *DEFA1*, *DEFA2 and DEFA3* (α-defensins 1, 2 and 3, respectively) were also observed to be highly downregulated.

**Figure 4:**
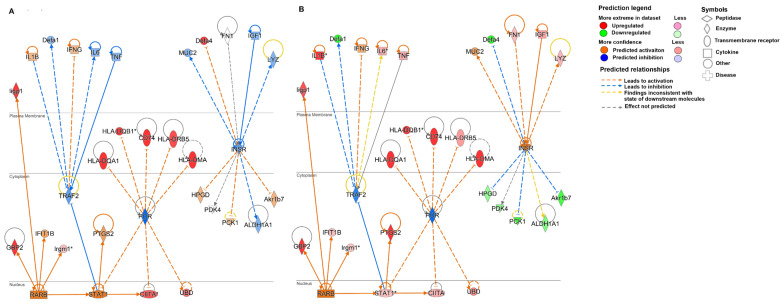
Gene network based analysis of role of α-defensins in *C. parvum* infection regulated by *INSR* and *TRAF2*. Overlay of molecular network generated with experimental datasets (LogFC2 adj *p*-value 0.05) from (**A**) day 45 PI and (**B**) day 93 PI. (*) Indicates that multiple identifiers are present in the dataset file which map to a single gene in the global molecular network.

Using the tool “My Pathways” available in IPA, customized pathways were created for target molecules of interest including *DEFA4*, *DEFA1*, *CIITA* and *IIGP1*. Interferon-inducible GTPase 1 (*IIGP1*) and class II, major histocompatibility complex, transactivator (*CIITA*) have been selected to showcase the network of genes known to be involved in inhibition of parasitic infection. *Cryptosporidium* is speculated to circumvent this action. *DEFA1* and *DEFA4* have been selected to identify the network of genes regulating their expression considering α-defensins as potential novel targets of *C. parvum* to persist the infection. The network for each target molecule was built on the molecules and/or relationships available in the database exclusively from epithelial cells, small intestine and large intestine. Further, the developed pathways were overlaid with experimental datasets (Log FC2 adj *p*-value 0.05) at day 45 PI and day 93 PI to measure the expression level of the molecules. As a result of this analysis, *DEFA4* expression was observed to be indirectly regulated by intestinal epithelial insulin receptors (*INSR*). At day 45 PI, *INSR* is predicted to be inhibited which results in upregulation of *DEFA4* in intestinal epithelial cells (Figure 4A). However, at day 93 PI, we observe an increase in the expression of *IGF1* (insulin growth factor 1) which is predicted to activate *INSR*, resulting in downregulation of *DEFA4* expression. Predicted activation of *INSR* is also supported by downregulation of other genes from the experimental dataset observed in the network such as hydroxyprostaglandin dehydrogenase (*HPGD*), pyruvate dehydrogenase (*PDK4*), *PCK1* (phosphoenolpyruvate carboxykinase 1), aldehyde dehydrogenase family member A 1 (*ALDH1A1*) and aldoketoreductase family 1 member B7 (*Akr1b7*) (Figure 4B). The second hypothetical network predicted *DEFA1* expression to be regulated by TNF receptor associated factor 2 (*TRAF2*). At day 45 PI, *DEFA1* was predicted to be downregulated with very low confidence score due to predicted inhibition of *TRAF2* (Figure 4A). However, at day 93 PI, with the increase in the expression levels of cytokines such as Interleukin 1β (*IL1β*) and *TNF*, *DEFA1* was predicted to be downregulated by *TRAF2* with a high confidence score (Figure 4B). Apart from the cytokines, a transcriptional factor such as Signal Transducer and Activator of Transcription 1 (*STAT1*) [z-score = 3.343] was also predicted to inhibit *TRAF2* (Figure 4). In IECs, *STAT1* expression is dependent upon the transcription factor retinoic acid receptor β (*RARβ*) [z-score = 2.363]. Moreover, upregulation of several immune response molecules such as *IIGP1*, *IGTP*, *GBP2* and *IRGM1* in the IECs was predicted to be dependent upon *RARβ* (Figure 3). In addition, *STAT,* is also regulated by one of the highly inhibited upstream regulators called *POR* [z-score = −2.813]. Downregulation of POR enzymes has been responsible for enhancing the expression of *STAT1* which is an activator of the class II transactivator for MHC II gene expression IECs (Figure 4). Thus, *POR* is indirectly responsible for regulation of expression of *CIITA* along with the molecules of MHC II group such as *CD74*, *HLADQA1*, *HLADMA*, *HLADQB1* and *HLADRB5* previously identified as genes responsible for inhibition of parasite infection.

### 3.4. Chronic Cryptosporidiosis Induces Tumor Microenvironment

**Figure 5:**
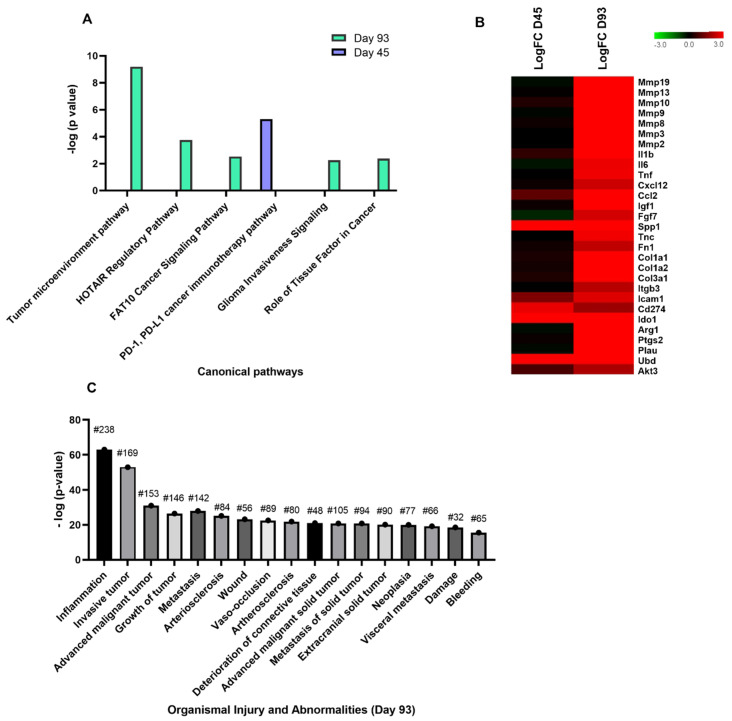
IPA analysis of tumor microenvironment induced by *C. parvum* infection. (**A**) Cancer related canonical pathways observed at days 45 and 93 PI are represented in terms of -log (*p*-value) on y-axis. (**B**) Heatmap of genes identified in tumor microenvironment canonical pathway at days 45 and 93 PI. (**C**) Cancer-related functions associated with “Disease/Disorder—Organismal Injury and Abnormalities” are represented in terms of −log (*p*-value) on y-axis. # = no. of differentially expressed genes.

Experimental datasets from days 45 and 93 PI were subjected to comparative IPA analysis to identify enriched “Canonical Pathways” associated with cancer by applying the -log(*p*-value) > 2 threshold. As a result, cancer related canonical pathways were majorly identified in the dataset at day 93 PI compared to day 45 PI (Figure 5A). “*PD-L1* Cancer Immunotherapy Pathway” (*p*-value = 3.02 × 10^−6^) was the only pathway identified within the dataset at day 45 PI. However, this pathway was predicted to be inhibited with an activation z-score of −2.0. Programmed cell death ligand-1 (*PD-L1*), also known as *CD274*, is an immune checkpoint molecule which is expressed by tumor cells or infiltrating myeloid cells to induce apoptosis of T cells and drive immune suppression which is highly expressed at day 45 PI (Log FC = 2.78).

The top ranked “Tumor Microenvironment Pathway” (*p*-value = 6.47 × 10^−10^) was predicted to be activated with an activation z-score of 5.099 at day 93 PI. This pathway had an overlap score of 15.6% with 29/173 molecules found within the dataset. A heatmap was generated to identify the regulation of expression level of these overlapping genes. All the genes identified to activate the tumor microenvironment pathway were highly upregulated at day 93 PI except secreted phosphoprotein 1 (*SPP1*), Indoleamine 2,3-dioxygenase 1 (*IDO1*), *UBD* and *CD274,* which were also highly upregulated at day 45 PI (Figure 5B). The other major pathways identified in terms of regulated gene numbers were HOX antisense intergenic RNA) regulatory pathway (HOTAIR) and *HLA*-*F adjacent transcript 10*, also known as UBD) cancer signaling pathway (FAT10). The identification of other genes from the datasets that are associated with canonical pathways such as “Glioma Invasiveness Signaling Pathway” and “Role of Tissue Factor in Cancer” highlighted the invasive nature of the tumor development.

This result was further confirmed by the identification of “Organismal Injury and Abnormalities” as one of the highly regulated disease/disorder (Figure 2B). After applying a z-score > 2 as threshold, it was observed that most of the cellular functions that define this disease/disorder are associated with cancer. The heatmap generated for “Organismal Injury and Abnormalities”, out of the 17 activated cellular functions with z-score > 2, 10 functions were associated with growth, metastasis, invasion and neoplasia of tumor. These 17 cellular functions are represented in terms of -log *p* values in Figure 5C. Invasive tumor (*p*-value = 9.37 × 10^−54^) had the highest activation z-score of 4.013 with a total of 169 molecules involved in the dataset (Figure 5C).

**Figure 6:**
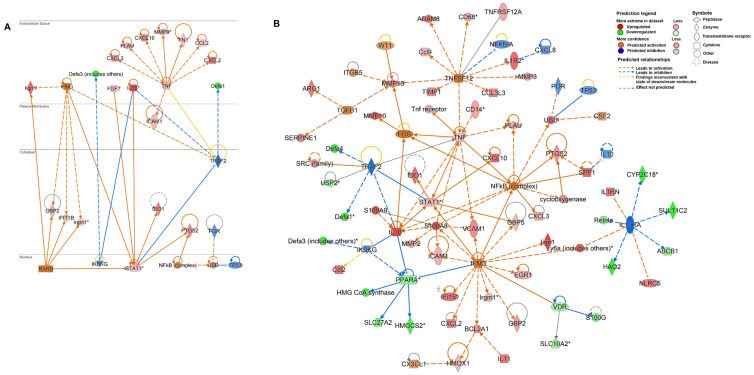
(**A**) Gene network based analysis of tumor associated genes in intestinal epithelial cells at day 93. (**B**) Customized gene network predicted within the tumor microenvironment induced by *C. parvum* infection. Overlay of molecular network is generated with experimental datasets from day 93 PI. (*) Indicates that multiple identifiers are present in the dataset file which map to a single gene in the global molecular network.

Using the tool “My Pathways”, customized pathways were created to target molecules of interest (*TNF* and *STAT1*). The network for each target molecule was built on the molecules and/or relationships available in the databases exclusively from epithelial cells or cancerous epithelial cell lines. Further, the developed pathways were overlayed with experimental datasets (LogFC2 adj *p*-value 0.05) obtained at day 93 PI to detect the expression levels of the predicted molecules. As shown in Figure 6A, *TNF-α* expression was observed to upregulate expression of several cancer-associated genes in the epithelial cells which include extracellular matrix (ECM) remodeling genes such as *MMP9*, *PLAU*, *FN1* and *ICAM1*, chemokines such as *CCL2*, *CXCL2*, *CXCL3* and *CXCL10* and regulatory genes such as *UBD* and *PTGS2*. In IECs, expression of *STAT1* gene was also predicted to be upregulated by inflammatory cytokines *IL1β* and *IFNγ* and growth factor *FGF7* identified as key molecules involved in tumor microenvironment. Moreover, activation of *STAT1* has been predicted to also upregulate expression of cancer associated genes such as *IDO1* and *PTGS2* (Figure 6A).

Figure 6B illustrates a more detailed gene network predicted to be involved in the tumor microenvironment induced by *C. parvum* infection. Upregulated expression of cytokines and chemokines indicated functionality of tumor-associated macrophages within the tumor microenvironment. These cytokines have been identified to be directly or indirectly responsible for cancer progression through remodeling of ECM by upregulating the expression of molecules such as *MMP2*, *MMP3*, *MMP9*, *MMP10*, *MMP13*, *PLAU*, *ICAM1* and *VCAM1*. Upregulated expression of *SPP1* has been identified as a candidate to trigger the release of these MMPs to induce metastasis of cancer cells via *NF-κβ*. *NF-κβ* has also been observed as central regulatory molecule as it is predicted to be activated by most of the pro-inflammatory cytokines such as *TNF-α*, *IFNγ* and *IL-β*. Its activation results in the upregulated expression of enzyme involved in synthesis of prostaglandins (*PTGS2*), *UBD*, chemokine (*CXCL3*), serine proteases (*PLAU*) and metalloproteinases (*MMP10* and *MMP13*).

Activated expression of *UBD* has been predicted to downregulate the p53 tumor-suppressor gene. Along with *NF-κβ*, *STAT1* is another transcription factor observed to be central to *IFNγ*, *TNF-α* and *IL-1β* mediated signaling. The network identified immunosuppressive cell signaling molecules such as *IDO1* to be regulated by *STAT1.* Anti-microbial peptides such as *DEFA1* and *DEFA4* were predicted to be downregulated by *STAT1* signaling whereas *DEFA3* appeared to be downregulated due to inhibition of *IKBKG* (inhibitor of nuclear factor kappa B kinase regulatory subunit gamma) caused by overexpression of *IL1β*. By inducing upregulation of *IFNγ* regulated molecules such as *GBP5*, *STAT1* expression can be considered to play a role in predicted activation of *IFNγ* along with other immune response molecules associated with parasitic infection such as *IIGP1*, *GBP2*, *GBP5*, *IRGM1* and *IFI16.* Activation of *IFNγ* can also be attributed to the detection of upregulated gene expression of Lymphocyte antigen 6 complex (*LY6C*), an antigen indicating the presence of inflammatory monocytes. Moreover, *IFNγ* along with *IL1β* was also predicted to negatively downregulate expression of nuclear receptor genes such as Vitamin D receptor (*VDR*) and Peroxisome Proliferator Activated Receptor Alpha (*PPARA*). These nuclear receptors especially *PPARA* in turn have been identified to negatively regulate the expression of tumor suppressor such as 3-Hydroxy-3-Methylglutaryl-CoA Synthase 2 (*HMGCS2*). The tumor microenvironment network also identified significant downregulation of genes associated with metabolism of hormones, drugs and xenobiotic compounds such as Cytochrome P450 Family 2 Subfamily C Member 18 (*CYP2C18*), *SLC10A2*, Solute Carrier Family 10, 27 Member 2 (*SLC27A2*), and Sulfotransferase Family 1C Member 2 (*SULT1C2*) which in turn is expected to inhibit *IL-10RA* (interleukin-10 receptor) expression. *IL-10*, an anti-inflammatory receptor is also predicted to be inhibited by immunosuppressor *SPP1*. Arginase1 (*ARG1*) is another immunosuppressive molecule identified in the network predicted to be regulated by *TGF-β* along with serine protease (*SERPINE1*) and metalloproteinase 13 (*MMP13*).

### 3.5. Validation of Target Genes by RT-qPCR

**Figure 7:**
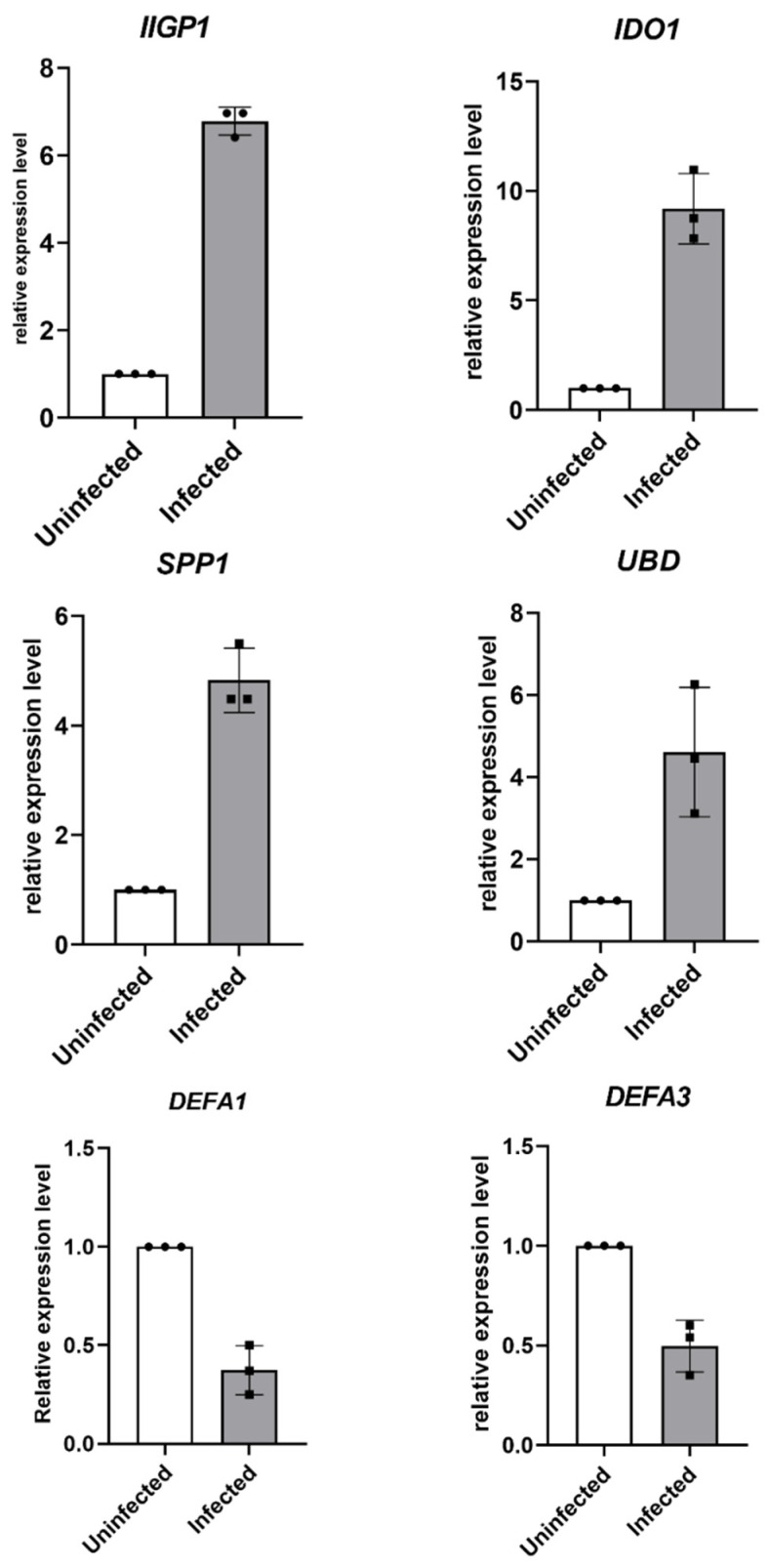
Validation of microarray data by RT-qPCR on a panel of seven targeted genes at day 93 PI. The expression levels were analyzed in triplicate and normalized to *GAPDH*. Each black circle and each black square indicates an individual uninfected and infected mouse respectively. The error bars indicate the mean with SD.

In total, six genes of interest were selected for RT-qPCR validation of microarray results at day 93 PI. These genes were chosen based on individual function speculated for them during *C. parvum* infection induced tumor development. *IIGP1* was chosen to validate the microarray results concerning the host immune defense against parasite infection, *IDO1*, *SPP-1* and *UBD* for the progression of tumor microenvironment, and *DEFA1* and *DEFA3* for the anti-microbial response speculated to be suppressed by *C. parvum* in order to propagate cancer. RT-qPCR analysis detected an average of 6.93, 7.83, 5.5 and 4.6 relative fold increases in the respective expression of *IIGP1*, *IDO1*, *SPP1* and *UBD* after normalization to the endogenous expression of *GAPDH* in the *C. parvum* infected caecum tissue. *DEFA1* and *DEFA3* expressions were observed to be decreased by a fold change of 0.5 (Figure 7).

## 4. Discussion

In the current study, a transcriptomic approach was conducted to explore the signaling pathways potentially involved in the development of neoplasia induced by *C. parvum*. Global upregulation of genes associated with the innate immune response was observed despite the administration to the animals with the anti-inflammatory and immunosuppressive drug, dexamethasone. The total number of genes subjected to core analysis by IPA was 37 genes for day 45 PI compared to 900 genes for day 93 PI. This difference in the number of differentially expressed genes per dataset could be attributed to the fact that it was possible to visualize the polypoid masses within the ileocecal region at day 93 PI before dissection for microarray analysis. In contrast, macroscopical lesions were not visible at day 45 PI and the samples were dissected blindly. The core analysis of the datasets focused upon the following modules namely upstream regulators, disease and functions and regulator effects. The aim of IPA upstream regulator analytic is to identify the cascade of upstream transcriptional regulators that can explain the gene expression changes in the experimental datasets and in turn help to clarify the biological activities occurring in the tissue. The top three common upstream regulators (*LPS*, *IFNγ* and *TNF-α*) identified at day 45 PI and day 93 PI, are likely obvious consequences of the presence of *C. parvum* infection in the tissue. Indeed, the prevailing transcriptional response of IECs to *C. parvum* was to upregulate gene targets of *IFNγ* signaling [36]. Moreover, *TNF-α* released from inflammatory monocytes assists *C. parvum* in loss of intestinal barrier to propagate the infection [20]. In parallel, *LPS* from bacteria are known activators of Toll-like receptor 4 (*TLR4*) signaling which is similarly activated for *C. parvum* eradication after activation of the *NF-κβ* signaling pathway [37]. Thus, identification of *LPS* as one of the upstream regulators suggested the presence of still unknown parasitic virulence factors homologous to *LPS*. The top “diseases and disorders” associated with the experimental datasets were “inflammatory response”, “immunological disease” and “organismal injury and abnormalities”. Moreover, identification of gastrointestinal disease as one of the disorders supports the fact that the differentially expressed genes were detected as a response to *C. parvum*. “Mammalian infection” was the top regulatory pathway identified to be inhibited at day 45 PI and the genes responsible for this action were associated with MHC class II molecules. Antigen presenting cells such as conventional dendritic cells and macrophages found in the intestinal lamina propria have been identified in response to *C. parvum* infection [38]. In IECs, *IFNγ* triggers *CIITA*, which in turn is responsible for the expression of MHC class II molecules [39].

Guanylate binding proteins (*GBP2*, *GBP4*, *GBP6*, *GBP8* and *GBP11*), a superfamily of large GTPases, were found upregulated. It is known that they are induced by *IFNγ* as a host response to external pathogens [22]. Especially *GBP2* has been identified to be recruited by another Apicomplexa, *Toxoplasma gondii* at the host-parasite interface, in the parasitophorous vacuole [40]. Considering, that the parasitophorous vacuole formation is also a hallmark feature of *Cryptosporidium* spp. infection, it may be suggested that upregulation of these genes represents a conserved mechanism of defense among Apicomplexan parasites. Similar observation has also been recorded in another microarray study on *C. parvum* infected intestinal epithelial cells of piglets [36]. On the basis of these data, the *IFNγ*-induced signaling pathway appears to be a highly enriched network on which the host defense mechanism depends.

In parallel to the host defense mechanism aiming at eradicating the infection, the parasite has developed several escape mechanisms to delay these protective mechanisms [38]. Particularly, it has been shown that *C. parvum* can circumvent *CCL20*, a chemokine known to exert anti-microbial activity [41]. Interestingly, this protection during infection was also downregulated at day 93 PI in our study. In addition, defensin-α (*DEFA4*) was highly upregulated at day 45 PI compared to day 93 PI wherein it was highly downregulated. α-defensin is a microbicidal peptide expressed by the Paneth cells to contribute to the innate enteric immunity which has shown parasiticidal activity against *Giardia intestinalis* [42]. Increased expression of LL37 and α-defensin 2 in response to IL18 perturbates intracellular development of *C. parvum* in human cell lines [43]. Thus, it can be proposed that α-defensins are part of the strategy of the host to eradicate *Cryptosporidium* infection even if the parasite seems to be able to downregulate these genes as another immune evasion strategy. Consistently, the downregulation of defensin-β (*DEFB1*) genes in host cells following *C. parvum* infection has been described to be the result of trans-suppression caused by an RNA transcript delivered into the host cell by the parasite [44]. *C. parvum* has been predicted to downregulate the expression of *DEFA4* via the *IGF* signaling pathway at day 93 PI. *IGF* signaling pathway has in turn been predicted to be activated by overexpression of *IGF1*. *IGF-1* signaling pathway was also observed to mediate downregulation of *HPGD* along with other dehydrogenases at day 93 PI, an enzyme responsible for degradation of prostaglandins which are produced by Prostaglandin-Endoperoxide Synthase 2 (*PTGS2*). Prostaglandins are pro-inflammatory lipid mediators which promote cancer cell proliferation, angiogenesis, survival, migration, and invasion [45]. Therefore, persistent *C. parvum* infection might possibly result in the attenuated expression of defensins with deregulation of the host immunity and alteration of the balance towards inflammation associated cancer. In parallel, loss of the *MCC* gene in a mouse model showed upregulation of *IFNγ*-induced GTPase superfamily in the absence of any external pathogen involvement in the inflamed colon and was restricted to immediate proximity of the damaged epithelial barrier leading to development of cancer [22]. Hence, it can be suggested that intestinal epithelial cells express *IIGP1* as an immune response against *C. parvum* but the parasite would be able to resist to this innate immune response. Then, the overexpression of *IIGP1* would result in chronic inflammation contributing to development of cancer.

The role of chronic cryptosporidiosis in the induction of digestive cancer was further confirmed after the analysis of molecular data at day 93 PI which allowed the identification of genes belonging to five canonical pathways associated with cancer. Moreover, the top disorder associated with this dataset termed as “Organismal Injuries and Abnormalities” was a result of several functions related to cancer progression such as neoplasia, growth, invasion, and metastasis of solid tumor. Identification of 29 out of 173 canonical molecules associated with “Tumor Microenvironment Pathway” predicts that *C. parvum* infection induces a pro-tumorigenic immune response, mediated by diverse immunosuppressive cell signaling molecules resulting in host immune evasion. Considering its over expression, *TNF-α* was identified as a key pro-inflammatory cytokine which stimulate tumor progression at day 93 PI. *TNF-α* produced by inflammatory monocytes assist *C. parvum* to alter the intestinal barrier and the intestinal permeability [20]. In a previous experimental animal infection with *C. parvum*, immunohistochemical abnormal localization of Wnt signaling pathway components together with alterations in the ultrastructure of adherens junctions of the ileo-cecal neoplastic epithelia were recorded [18]. Consequently, in *C. parvum* induced carcinogenesis, *TNF-α* could be an inflammatory stimuli triggering EMT and contributing to lesion development. In addition, expression of molecules involved in ECM remodeling such as several metalloproteinases (*MMP2*, *MMP9*, *MMP10* and *MMP13*), serine protease (*PLAU*) collagens, fibronectins and integrins indicate that functional EMT transition and metastasis take place within the tumor microenvironment [46].

On the other hand, oncogenic pathogens such as *H. pylori* or Epstein Barr virus share common signaling pathways which lead to EMT suggesting that these pathogens may be considered as EMT inducers able to cause a sustained activation of EMT regulating signaling pathways such as NFκβ, MAPK and PI3K/AKT [47]. Consistently, *AKT3*, one of the isoforms of *AKT* that modulates various cellular responses via PI3K/AKT pathway was upregulated at day 93 PI. Interestingly, the over-activation of *AKT* has also been reported in various cancers [48].

Inflammatory stimuli-mediated EMT has been shown to confer immunoregulatory properties to neoplastic epithelial cells by activation of *IDO1* and enhancing tumor immune escape [49]. Indeed, *IDO1* is responsible for catabolism of tryptophan within a tumor microenvironment. Unavailability of the tryptophan arrests T cell proliferation and induces apoptosis which results in immune escape of the cancer cells [50]. In accordance with these observations, we propose herein a gene network in which inflammatory EMT triggered by *IFNγ* and *TNF-α* via *STAT1* results in upregulation of *IDO1* in IECs. In support of this result, *IDO1* expression in Paneth cells has been observed to be strictly regulated by *STAT1* and regarded as an immunosurveillance escape strategy of colorectal cancer [51]. Even though, the downregulation of *IDO1* through the disruption of the *IFNγ* induced *STAT1* pathway has been reported in vitro in early stages of *C. parvum* infection of epithelial cells [52]. Within a tumor microenvironment, high expression of *IDO1* is also indicative of the presence of a population of tumor-infiltrating immune cells such as myeloid-derived suppressor cells (MDSCs) which are known to have suppressive effect on adaptive immune responses [50]. The presence of MDSCs in tumor microenvironment induced by *C. parvum* can also be predicted by the detection of upregulated expression of *ARG1*, another immune suppressive factor which depletes arginine in the microenvironment [50].

Upregulated expression of growth factors such as *IGF1*, *FGF7*, proteases such as *MMP2*, ECM constituents such as *SPP1* also known as osteopontin and chemokine such as *CXCL12* may indicate the presence of cancer-associated fibroblasts (CAFs) within the tumor microenvironment [53]. *FGF* and *CXCL12* released by CAFs are known to induce tumor growth and angiogenesis. Tumor-associated macrophages (TAMs) polarization represents a key process in tumor progression since TAMs are derived from blood monocytes that can be activated to either M1 (anti-tumor) or M2 (pro-tumor) polarization states depending on the microenvironment stimuli. Within the tumor microenvironment induced by *C. parvum*, upregulated expression of immune suppressive factors such as *ARG1* [54] and *SPP1* [55] probably indicates the presence of tumor promoting M2 TAMs. Inflammation caused by TAMs promotes tumor growth by inducing vascular permeability via upregulation of pro-inflammatory prostaglandins. Upregulated expression of *PTGS2*, previously described to be expressed in IECs can be predicted to increase prostaglandin production and assist TAMs in promoting tumor angiogenesis [56]. *STAT1* and *NF-κβ* have been identified as key transcriptional regulators of *PTGS2* expression, similar to what happens in *H. pylori* infection that induces gastric cancer via chronic inflammation that activates *NF-κβ* which in turn induces pro-inflammatory mediators such as *IL1, IL6, IL8, TNF-α* and *PTGS2* [57]. *UBD* which also contributes to colon cancer progression [58] was observed to be upregulated in the datasets and its expression was predicted to be induced by *TNF-α* in transformed epithelial cells. Moreover, IECs infected with *C. parvum* showed upregulated expression of *UBD* along with interferon stimulated genes [36]. Thus, it can be speculated that an inflammatory response induces the expression of *UBD* and its role in *C. parvum* associated oncogenesis can be explained by its ability to inhibit tumor suppressor *p53*. Consistently, after infection of animals with *C. parvum* an abnormal localization of p53 protein was found [18].

Even though the majority of the genes required for the maintenance of tumor microenvironment were detected at day 93 PI, some others genes such as *PD-L1*, an immune checkpoint gene, were observed to be exclusively expressed at day 45 PI. Indeed, *PD-L1* which is expressed on tumor cells and infiltrating myeloid cells can inhibit T cell function by inducing apoptosis [59]. Thus, *PD-L1* signaling could be predicted to mediate the contribution of chronic inflammation to carcinogenesis by preventing transformed epithelial cells from CD8^+^ T-cell attack at day 45 PI [57]. All these data suggests that the development of immunosuppressive tumor microenvironment would be associated with chronic cryptosporidiosis.

Along with all the upregulated genes, the list of several downregulated genes such as cytochrome p450 (*Cyp2c18*, *Cyp2c40*) sulfotransferases (*SULT1C2*), solute carrier proteins (*SLC10a2*, *SLC10A2*) also support the hypothesis of a role of persistent *C. parvum* infection leading to inflammation-assisted cancer progression. Downregulation of these genes was found in DSS-induced colitis model in mice [60] and a coordinated decrease in the expression of the drug response gene cluster was also observed in a mouse model which developed cancer as a result of inflammation-induced DNA damage [22].

## 5. Conclusions

The data collected herein strongly suggest that chronic inflammation associated with chronic infection plays an important role in *C. parvum*-induced digestive neoplasia. The ability of the parasite to evade the host innate immune response by resisting the upregulated expression of *IFNγ*-stimulated genes and downregulating expression of α-defensins gives rise to the chronic inflammation. Systematic inflammation may contribute to the *C. parvum*-induced immunosuppressive tumor microenvironment. Further studies are needed to understand the immune escape strategies and the pathogenicity of this parasite highly oncogenic when inoculated in an animal model, and to substantiate additional links with cancer development.

## Figures and Tables

**Table 1:** Altered expression of highly regulated genes at days 45 and 93 PI.

Gene Symbols and Names	Direction of Regulation	LogFC Day 45 PI	LogFC Day 93 PI
Iigp1; interferon inducible GTPase 1	Up	6.78	5.74
Defa4; defensin, alpha, 4	Up (day 45 PI) Down (day 93 PI)	6.37	−6.07
H2-DMb1;histocompatibility 2, class II, locus Mb1	Up	6.35	3.20
Tgtp2; T cell specific GTPase 2	Up	6.31	5.13
Cd74; CD74 antigen	Up	6.23	2.99
H2-Ab1; histocompatibility 2, class II antigen A, β	Up	6.21	2.91
H2-Eb1; histocompatibility 2, class II antigen E, β	Up	5.89	3.27
Ciita; class II transactivator	Up	5.63	2.73
H2-Aa; histocompatibility 2, class II antigen A, α	Up	5.31	2.56
UBD; ubiquitin D	Up	5.15	4.25
Ido1; indoleamine 2,3-dioxygenase 1	Up	5.07	4.41
Cxcl10; chemokine (C-X-C motif) ligand 10	Up	4.56	4.44
Gbp11; guanylate binding protein 11	Up	4.39	3.11
Cxcl9; chemokine (C-X-C motif) ligand 9	Up	4.38	3.68
Gbp4; guanylate binding protein 4	Up	4.14	2.81
Gbp2b; guanylate binding protein 2b	Up	3.90	2.93
Gbp6; guanylate binding protein 6	Up	3.90	3.20
Il18bp; interleukin 18 binding protein	Up	3.68	3.21
Gbp8; guanylate-binding protein 8	Up	3.60	2.06
Ly6a; lymphocyte antigen 6 complex, locus A	Up	3.35	5.52
Igtp; interferon gamma induced GTPase	Up	3.37	2.90
Gbp2; guanylate binding protein 2	Up	3.32	2.92
Spp1; secreted phosphoprotein 1	Up	3.04	5.07
Ly6e; lymphocyte antigen 6 complex, locus E	Up	2.13	5.25
S100a9; calgranulin B	Up	N/A ^a^	9.14
S100a8; calgranulin A	Up	N/A ^a^	8.42
Mmp10; matrix metallopeptidase 10	Up	N/A ^a^	6.76
Il1b; interleukin 1 beta	Up	N/A ^a^	6.74
Defa2; defensin, alpha, 2	Down	N/A ^a^	−6.02
Il1rl1; interleukin 1 receptor-like 1	Up	N/A ^a^	5.84
Ifi44l; interferon-induced protein 44 like	Up	N/A ^a^	5.77
Arg1; arginase	Up	N/A ^a^	5.34
Defa3; defensin, alpha, 3	Down	N/A ^a^	−5.76
Mmp8; matrix metallopeptidase 8	Up	N/A ^a^	5.74
Il11; interleukin 11	Up	N/A ^a^	5.74
Mmp13; matrix metallopeptidase 13	Up	N/A ^a^	5.49
Defa1; defensin, alpha 1	Down	N/A ^a^	−5.76
Ifi202b; interferon activated gene 202B	Up	N/A ^a^	5.27
Cxcl1; chemokine (C-X-C motif) ligand 1	Up	N/A ^a^	5.27
Ccl3; chemokine (C-C motif) ligand 3	Up	N/A ^a^	5.23
Ccl2; chemokine (C-C motif) ligand 2	Up	N/A ^a^	5.19
Slc37a2; solute carrier family 37 (glycerol-3-phosphate transporter), member 2	Down	N/A ^a^	−4.50
Col1a1; collagen, type I, alpha 1	Up	N/A ^a^	4.87
Il1r2; interleukin 1 receptor, type II	Up	N/A ^a^	4.95
Adam8; a dis integrin and metallopeptidase domain 8	Up	N/A ^a^	4.89
Cxcl5; chemokine (C-X-C motif) ligand 5	Up	N/A ^a^	4.87
Sult1c2; sulfotransferase family, cytosolic, 1C, member 2	Down	N/A ^a^	−4.68
Ifit2; interferon-induced protein with tetratricopeptide repeats 2	Up	N/A ^a^	4.63
Mmp3; matrix metallopeptidase 3	Up	N/A ^a^	4.58
Cyp2c40; cytochrome P450, family 2, subfamily c, polypeptide 40	Down	N/A ^a^	−4.33
Cxcl2; chemokine (C-X-C motif) ligand 2	Up	N/A ^a^	4.14
Col1a2; collagen, type I, alpha 2	Up	N/A ^a^	4.14
Ptgs2; prostaglandin-endoperoxide synthase 2	Up	N/A ^a^	4.04

N/A ^a^, not applicable.

## Data Availability

The data provided in this study has been deposited at NCBI Gene Expression Omnibus (GSE188591) and is available at https://www.ncbi.nlm.nih.gov/geo/query/acc.cgi?&acc=GSE188591 (accessed on 12 November 2021).

## Supplementary Materials

The following are available online at www.mdpi.com/article/10.3390/microorganisms9122569/s1, Table S1. Primers used for RT-qPCR. Table S2. Differentially regulated genes at day 45 PI (LogFC2.0; adj *p*-value < 0.05). Table S3. Differentially regulated genes at day 93 PI (LogFC2.0; adj *p*-value < 0.05). References [61,62,63,64] are cited in the Supplementary Materials.
