# Supplementary material for: Persistent Cryptosporidium parvum Infection Leads to the Development of the Tumor Microenvironment in an Experimental Mouse Model: Results of a Microarray Approach
Source: Microorganisms. 2021 Dec 12;9(12):2569. doi: 10.3390/microorganisms9122569 (PMC8704780; doi:10.3390/microorganisms9122569)
Supplement: Supplementary file 1 [file microorganisms-09-02569-s001.zip › Supplementary Table S1.pdf]

**Table S1 - Primers used for RT-qPCR**				
**Gene ID**	**Primer bank ID**	**Primers**	**Sequences**	**Product size (bp)**
IIGP1	11140831a1	F	5’ CAGGACATCCGCCTTAACTGT 3’	519
		R	5' AGGAAGTAAGTACCCATTAGCCA 3’	
IDO1	6680346c2	F	5’ GCCTCCTATTCTGTCTTATGCAG 3’	194
		R	5’ ATACAGTGGGGATTGCTTTGATT 3’	
SPP1	6678113a1	F	5’ AGCAAGAAACTCTTCCAAGCAA 3’	134
		R	5’ GTGAGATTCGTCAGATTCATCCG 3’	
UBD	12963519a1	F	5’ CCAATGGCGGTTAATGACCTT 3’	141
		R	5’ TTTCGATGGGGCTTGAGGATT 3’	
DEFA1	Ref.4	F	5’ TCCTCCTCTCTGCCCTTGTC 3’	205
		R	5’ CCTTTGCAGCCTCTTGATCT 3’	
DEFA3	Ref.4	F	5’ TAGTCCTCCTCTCTGCCCTC 3’	245
		R	5’ ATGACCCTTTCTGCAGGTCC 3’	
GAPDH	126012538c3	F	5’ TGGCCTTCCGTGTTCCTAC 3’	178
		R	5’ GAGTTGCTGTTGAAGTCGCA 3’	
